# Comparative transcriptome analysis reveals differentially expressed genes related to the tissue-specific accumulation of anthocyanins in pericarp and aleurone layer for maize

**DOI:** 10.1038/s41598-018-37697-y

**Published:** 2019-02-21

**Authors:** Tingchun Li, Wei Zhang, Huaying Yang, Qing Dong, Jie Ren, Honghong Fan, Xin Zhang, Yingbing Zhou

**Affiliations:** 1 Corn Research Center, Tobacco Research Institute, Anhui Academy of Agricultural Sciences, Hefei, 230031 P. R. China; 2 School of Life Sciences, Anhui Agricultural University, Hefei, 230036 P. R. China

**Keywords:** Plant physiology, Secondary metabolism

## Abstract

Purple corn is a rich source of anthocyanins. In the experiment, two anthocyanins-enriched purple corn lines Ha0414 and Ha6130 were identified. The anthocyanins were respectively accumulated in the pericarp of Ha0414 and the aleurone layer of Ha6130 with different composition and content. Transcriptome analysis of the two tissues in both lines identified 16 and 14 differentially expressed genes belonging to anthocyanin metabolism pathway in pericarp and the aleurone layer, individually. Of these genes, two genes encoding 2-oxoglutarate (2OG) and Fe (II)-dependent oxygenase superfamily proteins, and one gene annotated as UDP-glycosyltransferase superfamily protein exhibited increased transcript abundance in both the colored pericarp and aleurone layer. Otherwise, one gene annotated as flavonoid 3′, 5′-hydroxylase, and another gene encoding flavonoid 3′-monooxygenase displayed increased transcript abundance in the aleurone layer of Ha6130. Moreover, 36 transcription factors were identified with increased transcript abundance in the pericarp of Ha0414, such as bHLH transcription factors, WRKY transcription factors, and HB transcription factors. And 79 transcription factors were isolated with an increased expression level in the aleurone layer of Ha6130, including MYB transcription factors, MYB-related transcription factors, and bHLH transcription factors. These genes expression may result in the tissue-specific accumulation of anthocyanins in pericarp and aleurone layer.

## Introduction

Plant foods with high content of anthocyanins are usually appreciated for their health-protective benefits^1–4^. More and more researchers demonstrated that anthocyanins perform critical biological functions, including antioxidant activities, scavenging of free radical and inhibition of lipid peroxidation^2,3^. Anthocyanin is synthesized via the flavonoid pathway. Eight genes controlled its accumulation, including *cinnamate-4-hydroxylase (C4H)*, *4-coumarate*: *CoA ligase* (*4CL*), *chalcone synthase* (*CHS*), *chalcone isomerase* (*CHI*), *flavanone 3-hydroxylase* (*F3H*), *dihydroflavonol 4-reductase* (*DFR*), *anthocyanidin synthase* (*ANS*), *UDP-glucose: flavonoid 3-O-glucosyltransferase* (*UFGT*)^5^. Besides, transcription factors such as *MYB*, basic helix-loop-helix (*bHLH*), *WD40* and *WRKY* were reported to participate in the temporal and spatial control of expression of anthocyanin biosynthetic genes in the various tissues^6^. For example, *DcMYB6*, an R2R3-type *Myb* gene, was involved in regulating anthocyanin biosynthesis in purple carrot taproots^7^. Co-expression of *bHLH* and *Myb* induced anthocyanin biosynthesis in the hairy root of *Nicotiana tabacum* and *Ipomea tricolor* ^8^. Moreover, MYB–bHLH–WD repeat protein (MBW) complex has been verified to be responsible for the regulation of the anthocyanin metabolism pathway^9^. And WRKY can join the MBW complex to participate in the regulation of anthocyanin biosynthesis^10^.

Corn (*Zea mays*) is one kind anthocyanins-rich source in cereals^2,11^. Anthocyanins could be accumulated in its stalk, cob, leaf, and seed^12^. For the edible use, researchers pay more attention to the anthocyanins in the aleurone layer and pericarp of the seed. And various kinds of the colored seeds including blue, pink and purple have been identified^13,14^. Further chromatography analysis revealed that the pigments in the aleurone layer and pericarp belonged to anthocyanins^14^. The different components and contents of anthocyanins conferred the various kinds of the color on the seed^14^. Previous studies have isolated several pathway genes of anthocyanin in maize, such as *ZmCHS* (*c2*), *ZmDFR* (*a1*), *ZmANS* (*a2*), *ZmCHS* (*chi*), *ZmF3H* (*fht1*), *ZmF3*′*H* (*pr1*), *ZmUFGT* (*bz1*) and *ZmGST* (*bz2*) (Supplementary Table 1)^15,16^. Moreover, transcription factors including *ZmbHLH* (*Sn*, *r1*, *b1*), *ZmMYB* (*c1*, *p1*, *pl*) and *ZmWD40* (*pac1*) have been identified for its import role in transcriptional regulation of biosynthetic genes in anthocyanin metabolism pathway (Supplementary Table 1)^2^. The interaction of these transcription factors and their target genes resulted in both spatial and temporal biosynthesis of anthocyanins in maize seeds.

To our knowledge, most of the previous studies were more focused on single or several genes related to anthocyanin biosynthesis. In the experiment, two purple corn lines were identified with tissue-specific accumulations of anthocyanins in the aleurone layer and pericarp of seeds. The components and contents of anthocyanins were analyzed. Moreover, by using comparative transcriptome analysis, the differentially expressed genes related to the tissue-specific accumulation of anthocyanins were identified. The results would imply the molecular mechanism for anthocyanin biosynthesis in maize seeds.

## Results

### Tissue-specific accumulation of anthocyanins in the pericarp and aleurone layer

**Figure 1:**
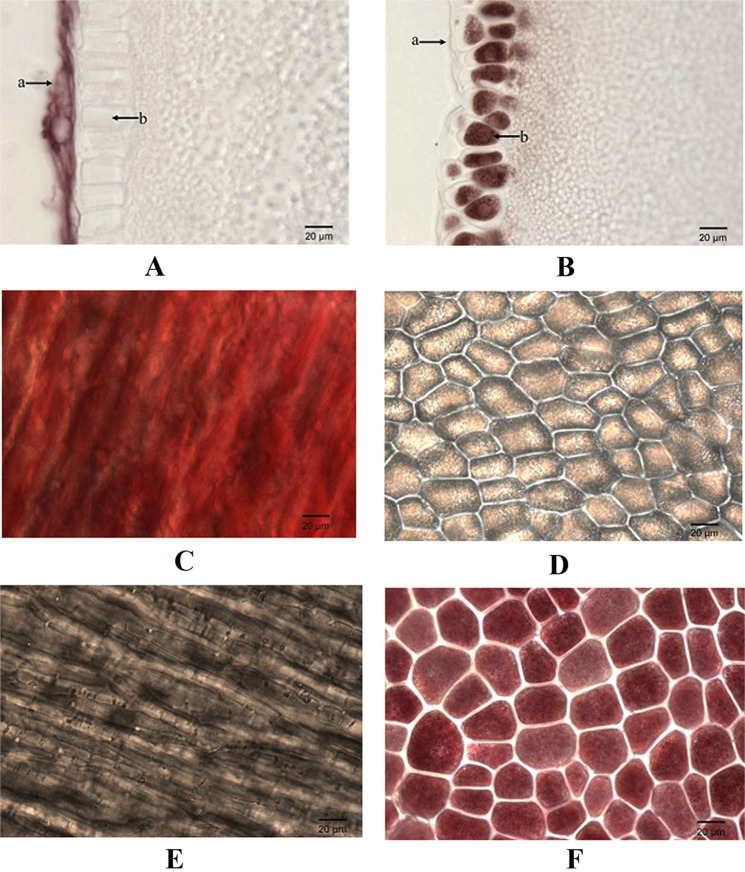
Microscopic observation of anthocyanin biosynthesis in pericarp and aleurone layer of Ha0414 and Ha6130. The letters (**A**) and (**B**) indicate the cross-section of seeds for Ha0414 and Ha6130, respectively. The letter “a” showed the pericarp. The letters (**C**) and (**E**) indicate the front side of the pericarp for Ha0414 and Ha6130, respectively. The letters (**D**) and (**F**) show the front side of the aleurone layers for Ha0414 and Ha6130, respectively.

To identify the anthocyanin-pigmented tissues, the immature seeds at 25 DPP were collected and fixed with FAA fixative. By cross-section with a microtome, the microstructure was observed. As shown in Fig. 1, anthocyanins were only accumulated in the pericarp of Ha0414 and the aleurone layer of Ha6130. There were no anthocyanins in the pericarp of Ha6130 and the aleurone layer of Ha0414. Moreover, by using an anatomical lens, the fresh pericarp and aleurone layer were dissected and separated from the seed at 25 DPP. The microscopic observation further confirmed that anthocyanins only accumulated in the pericarp of Ha0414 and the aleurone layer of Ha6130.

### Quantitative and qualitative analysis of anthocyanins in the pericarp and aleurone layer

**Figure 2:**
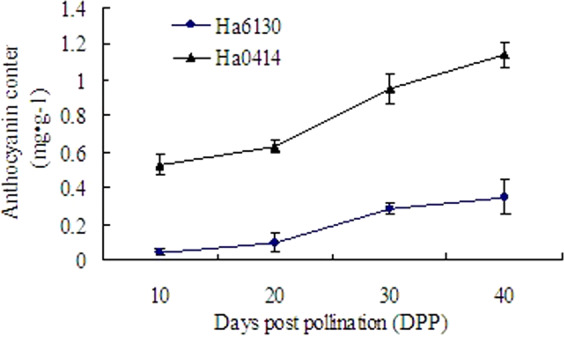
Anthocyanins content in seeds of Ha0414 and Ha6130.

As shown in Fig. 2, the contents of anthocyanins gradually increased during the development stages from 10 to 40 DPP. And the rapid accumulation of anthocyanins was at the later development stages after 25 DPP. Otherwise, the anthocyanins contents in the pericarp of Ha0414 were more than that in the aleurone layer of Ha6130.

**Figure 3:**
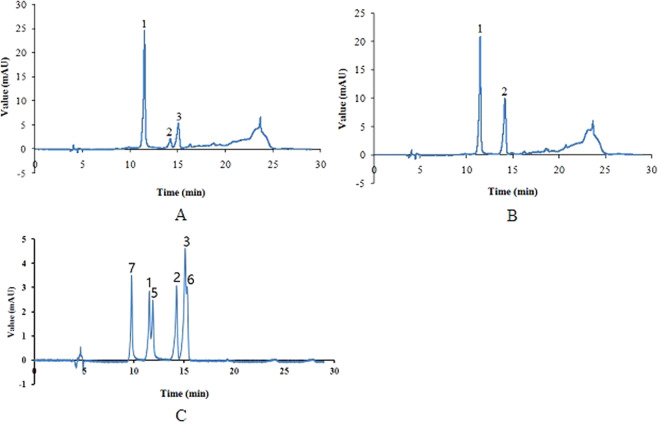
Analysis of anthocyanins components in the pericarp of Ha0414 and aleurone layer of Ha6130 by high-performance liquid chromatography at 520 nm. The letter (**A**) indicated the components of anthocyanin in pericarp for Ha0414. The letter (**B**) showed the ingredients of anthocyanin in the aleurone layer for Ha6130. The letter (**C**) stated the standards of anthocyanins ingredients, which included Cyanidin-3-glucoside (1), Pelargonidin-3-glucoside (2), Peonidin-3-glucoside (3), Petunidin-3-glucoside (4), Malvidin 3,5-diglucoside (5), delphinidin-3-glucoside (6).

To analyze the composition of anthocyanins in the pericarp and aleurone layer, high-performance liquid chromatography was employed to detect the components of the anthocyanins. The results showed that three main ingredients were detected in the pericarp of Ha0414, including cyanidin-3-glucoside (1), pelargonidin 3-glucoside (2), and peonidin-3-glucoside (3) (Fig. 3). And two ingredients were identified in the aleurone layer of Ha6130 including cyanidin-3-glucoside (1) and pelargonidin 3-glucoside (2). So, the main components of anthocyanins including cyanidin-3-glucoside and pelargonidin-3-glucoside are the same in the pericarp and aleurone layer. Nevertheless, the component peonidin-3-glucoside was only detected in the pericarp of Ha0414.

### Production of RNA-seq and mapping of reads to the B73 reference genome

To identify tissue-specifically expressed genes related to anthocyanin biosynthesis, total RNA with high quality was pooled from the pericarp and aleurone layer for transcriptome analysis. By using Illumina sequencing with the HiSeq 2500 platform, the raw data of the pericarp and aleurone layer for both Ha0414 and Ha6130 were obtained (Table 1). For Ha0414, it contained 9.08G clean bases read with 57.17% GC ratio for the aleurone layer and 8.09G clean bases with 59.24% GC contents for the pericarp. For Ha6130, it included 7.76G clean bases with 57.25% GC proportion for the aleurone layer and 8.6 G clean bases with 59.64% GC ratios for the pericarp. Besides, after alignment to B73 maize genome using the Tophat software, the percentage of total mapped reads account for 66.76%, 62.68%, 65.91% and 55.81% for the aleurone layer of Ha0414 (Ha0414A), the pericarp of Ha0414 (Ha0141P), the aleurone layer of Ha6130 (Ha6130A), and the pericarp of Ha6130 (Ha6130P), respectively (Table 1). Accordingly, the ratios of uniquely mapped reads reached 65.34%, 61.12%, 64.02%, and 54.16%, individually. Moreover, the correlation analysis indicated that the same tissue has the higher value of correlation coefficient, which reached 0.818 for the aleurone layer and 0.842 for the pericarp between Ha0414 and Ha6130 (Supplementary Fig. 1). Nevertheless, the correlation ratios were 0.531 and 0.447 between the pericarp and aleurone layer for two lines. These results may imply the tissue-specific expression of unigenes.

**Table 1:** Summary of data generated in the transcriptome sequence of maize.

Sample name	Ha0414A	Ha0414P	Ha6130A	Ha6130P
Raw reads	62584940	63132140	59658254	60811336
Clean reads	60539918	53931136	51714328	57344006
clean bases	9.08 G	8.09 G	7.76 G	8.6 G
Error rate (%)	0.02	0.02	0.02	0.02
Q20 (%)	95.71	96.26	96.16	95.01
GC content (%)	57.17	59.24	57.25	59.64
Total mapped reads	40417387	33802169	34087425	32002174
Percentage of total mapped reads (%)	66.76	62.68	65.91	55.81
Uniquely mapped reads	39556648	32960221	33107893	31058511
Percentage of uniquely mapped reads (%)	65.34	61.12	64.02	54.16
Multi-position mapped reads	860739	842948	979532	943663
Percentage of multi-position mapped reads (%)	1.42	1.56	1.89	1.65

Note: Percentage of total mapped reads shown ratios of reads that mapped to the B73 genome out of the total number of trimmed reads; Percentage of uniquely mapped reads implied percentage of uniquely mapped reads out of the total number of mapped reads.

### Screening and gene ontology classification of differentially expressed genes related to the tissue-specific accumulation of anthocyanins

**Figure 4:**
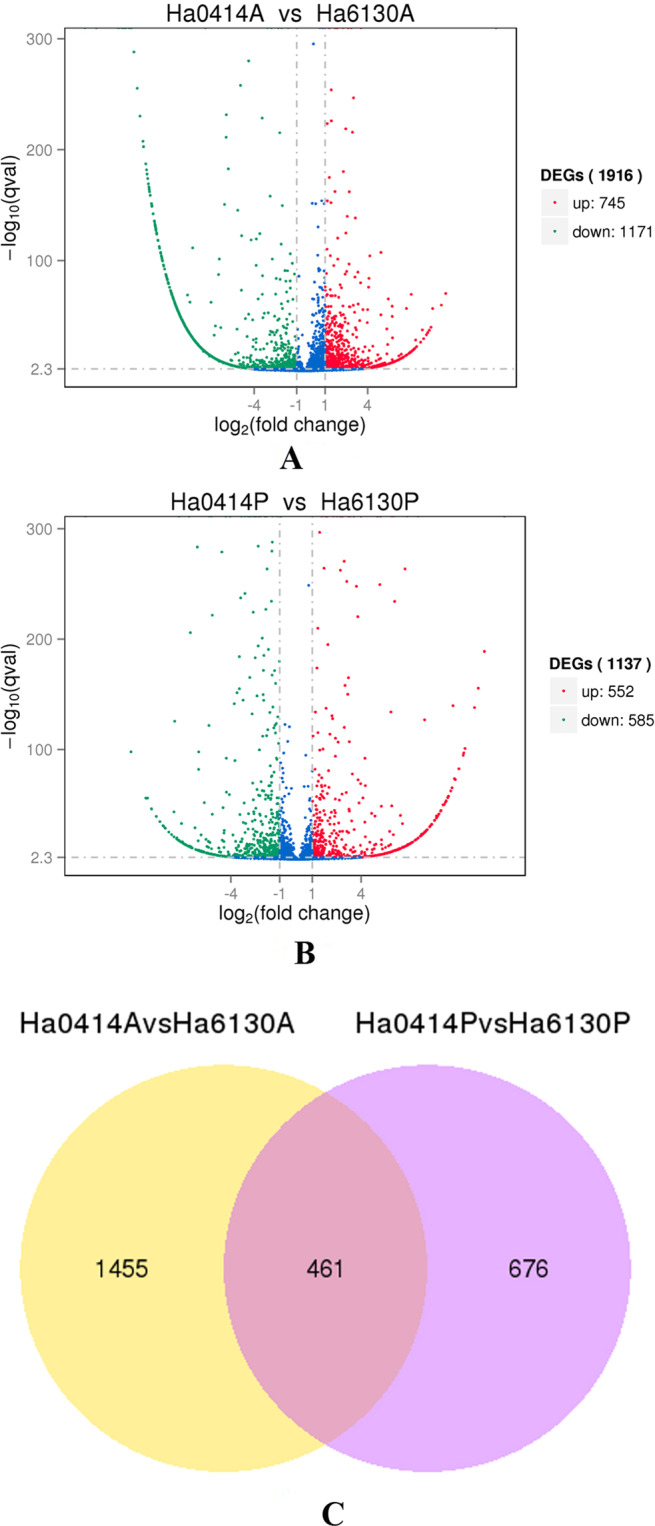
The distribution of differentially expressed genes related to the tissue-specific accumulation of anthocyanins in pericarp and aleurone layer. The letter (**A**) showed the differentially expressed genes in the aleurone layer between Ha0414 and Ha6130. The letter (**B**) referred to the differentially expressed genes in the pericarp between Ha0414 and Ha6130. The letter (**C**) indicated the co-expressed genes among the differentially expressed genes in pericarp and aleurone layer.

To obtain the differentially expressed genes related to the tissue-specific accumulation of anthocyanins, the transcriptomes were compared between the same tissues in Ha0414 and Ha6130. As a result, 1916 differentially expressed genes (DEGs) with 745 up-regulated genes and 1171 down-regulated genes were identified in the aleurone layer of Ha6130, if compared with that of Ha0414 (Fig. 4). And 1173 DEGs with 552 up-regulated genes and 585 down-regulated genes were isolated in the pericarp of Ha0414 in comparison with of Ha6130. Of these differentially expressed genes, 461 DEGs were identified with co-expression in both the aleurone layer of Ha6130 and pericarp of Ha0414. Besides, gene ontology classification was performed to analyze those genes functions. As a result, the proteins encoded by these genes were assigned to the biological process, cellular component and molecular functions such as single-organism metabolic process, secondary metabolic process, dioxygenase activity, 3-hydroxyacyl-CoA dehydrogenase activity, and so on.

### KEGG enrichment analysis of metabolic pathways for differentially expressed genes related to the tissue-specific accumulation of anthocyanin

**Figure 5:**
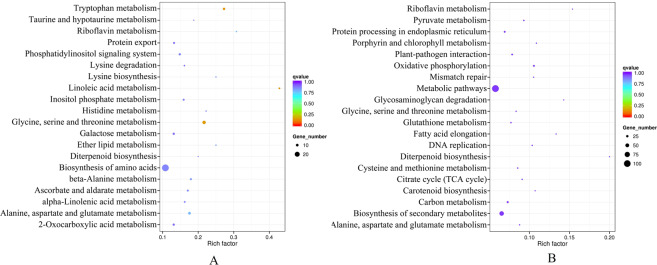
Statistical enrichment analysis of metabolic pathways for differentially expressed genes. The letter (**A**) indicated statistical enrichment analysis of differentially expressed genes in the aleurone layer between Ha0414 and Ha6130. The letter (**B**) indicated statistical enrichment analysis of differentially expressed genes in the pericarp between Ha0414 and Ha6130.

KEGG enrichment analyses of metabolic pathways revealed that 108 metabolic pathways were differentially regulated in the aleurone layer of Ha6130 (Fig. 5A). Among them, four metabolic pathways including oxidative phosphorylation with 12 genes, cysteine and methionine metabolism with 7 genes, pyruvate metabolism with 7 genes, and carbon metabolism with 13 genes were significantly up-regulated in aleurone layer of Ha6130 in comparison with that of Ha0414 (Data is not shown). Moreover, in contrast with the pericarp of Ha6130, there are 94 metabolic pathways were differentially regulated in the pericarp of Ha0414 (Fig. 5B). Among them, 27 genes belonging to five metabolic pathways were obviously up-regulated, which included 6 genes of tryptophan metabolism, 8 genes of glycine, serine and threonine metabolism, 7 genes of alanine, aspartate and glutamate metabolism, 3 genes of riboflavin metabolism, and 3 genes of diterpenoid biosynthesis.

### Analysis of differentially expressed genes related to anthocyanin biosynthesis

**Figure 6:**
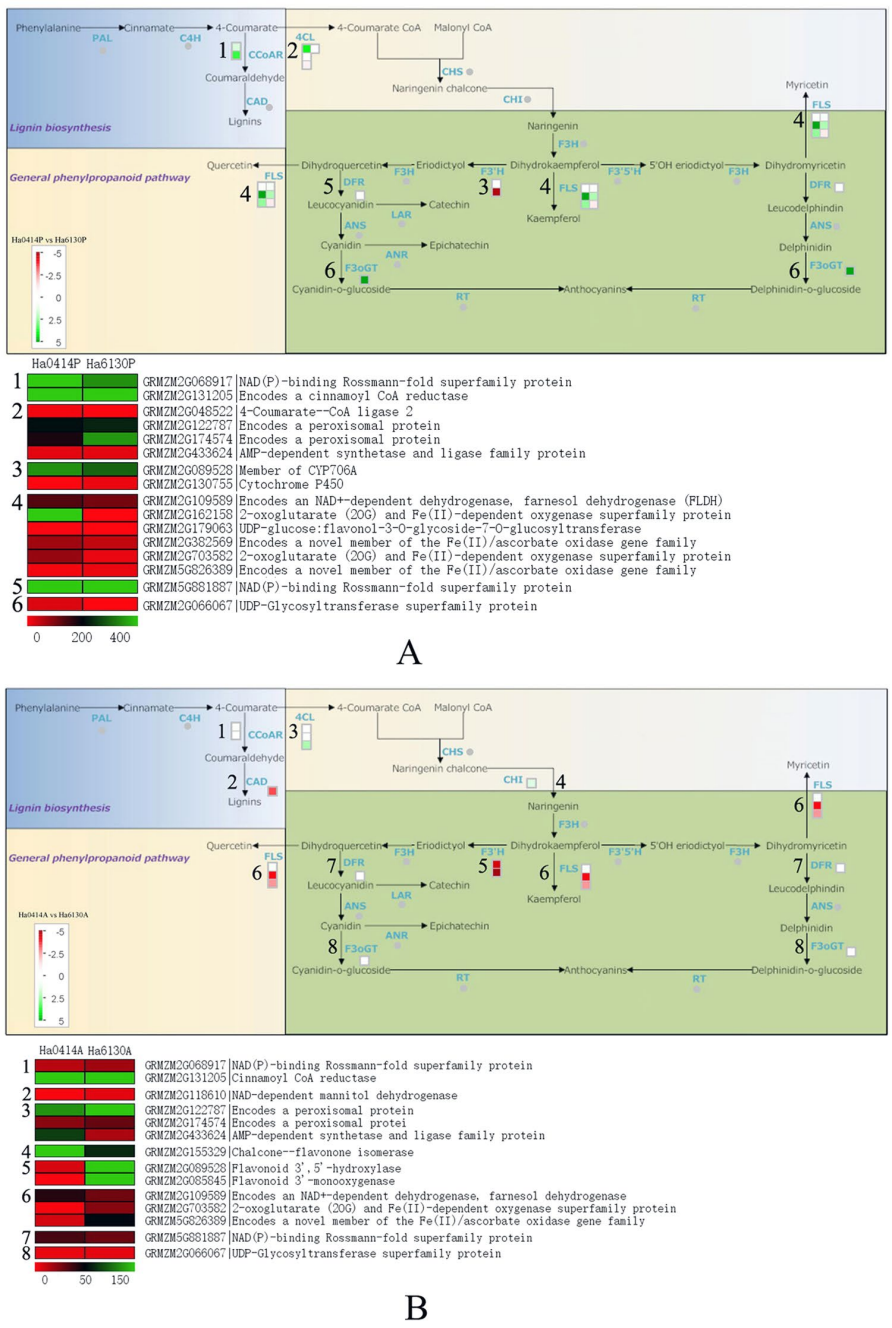
The differential expressed genes related to anthocyanin biosynthesis in the pericarp of Ha0414 and the aleurone layer of Ha6130. The letter (**A**) indicated the differential expressed genes related to anthocyanin biosynthesis in the pericarp of Ha0414.The letter (**B**) showed the differential expressed genes in relationship with anthocyanin biosynthesis in the aleurone layer of Ha6130.

As is shown in Fig. 6A, 16 genes from the anthocyanin biosynthesis pathway with different expression level in the pericarp were mapped to the metabolic pathway. Of these genes, 11 genes were isolated with increased transcript levels in the pericarp of Ha0414, if compared with that of Ha6130. The isoforms encoded by these genes included two of NAD(P)-binding Rossmann-fold superfamily protein (GRMZM2G068917 and GRMZM5G881887), one of cinnamoyl CoA reductase (GRMZM2G131205), one of 4-Coumarate-CoA ligase 2 (GRMZM2G048522), one of member of CYP706A protein (GRMZM2G089528), one of farnesol dehydrogenase (GRMZM2G109589), one of UDP-glucose:flavonol-3*-O-*glycoside-7*-O-*glucosyltransferase (GRMZM2G179063), two of 2-oxoglutarate (2OG) and Fe(II)-dependent oxygenase superfamily proteins (GRMZM2G162158 and GRMZM2G703582), one of Fe(II)/ascorbate oxidase gene family protein (GRMZM2G382569), and one of UDP-glycosyltransferase superfamily protein (GRMZM2G066067). Otherwise, 5 genes were identified with decreased transcript levels in the pericarp of Ha0414, including two of peroxisomal proteins (GRMZM2G122787 and GRMZM2G174574), one of AMP-dependent synthetase and ligase family protein (GRMZM2G433624), one of cytochrome P450 (GRMZM2G130755), and a novel member of the Fe(II)/ascorbate oxidase gene family (GRMZM5G826389). In contrast with the differentially expressed genes in the pericarp, 14 genes were identified with nine up-regulated and five down-regulated in the aleurone layer of Ha6130 if compared with that of Ha0414 (Fig. 6B). The isoforms encoded by the up-regulated genes included one of NAD(P)-binding Rossmann-fold superfamily protein (GRMZM2G068917), one of NAD-dependent mannitol dehydrogenase (GRMZM2G118610), two of peroxisomal proteins (GRMZM2G122787 and GRMZM2G174574), one of flavonoid 3′,5′-hydroxylase (GRMZM2G089528), one of flavonoid 3′-monooxygenase (GRMZM2G085845), one of 2-oxoglutarate (2OG) and Fe(II)-dependent oxygenase superfamily protein (GRMZM2G703582), one of novel member of the Fe(II)/ascorbate oxidase gene family (GRMZM5G826389), and one of UDP-glycosyltransferase superfamily protein (GRMZM2G066067).

**Figure 7:**
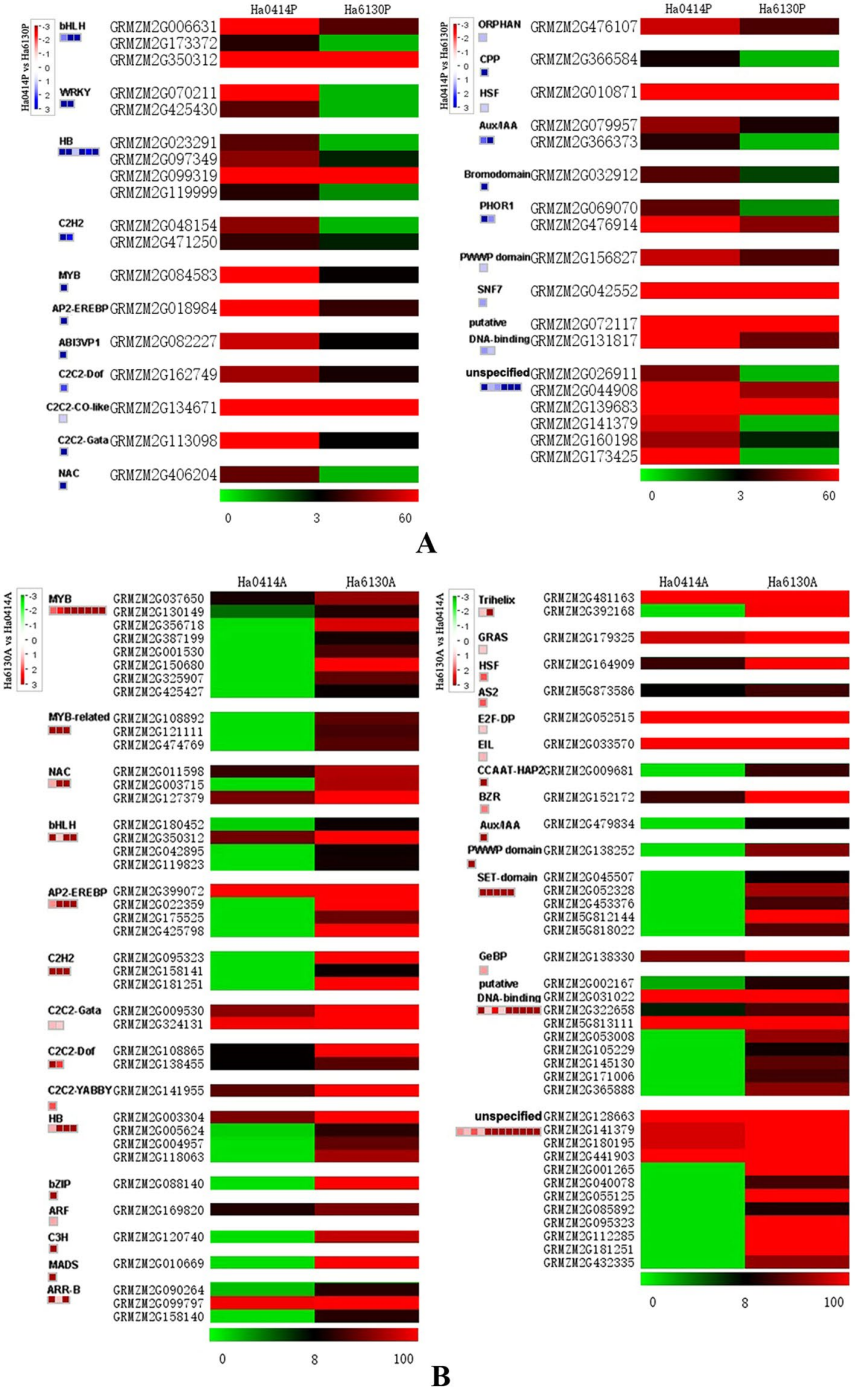
The up-regulated expression of transcription factors in the pericarp of Ha0414 and the aleurone layer of Ha6130. The letter (**A**) indicated the up-regulated expression of transcription factors in the pericarp of Ha0414 in comparison with that of Ha6130.The letter (**B**) showed the up-regulated expression of transcription factors in the aleurone layer of Ha6130 if compared with that of Ha0414.

Apart from anthocyanin pathway genes, transcription factors were involved in the tissue-specific biosynthesis of anthocyanins. As shown in Fig. 7A, 36 transcription factors were identified with increased transcript abundance in the pericarp of Ha0414 if compared with that of Ha6130. These transcription factors were distributed in 21 transcription factor families such as three of bHLH transcription factors (GRMZM2G005631, GRMZM2G173372, and GRMZM2G350312), two of WRKY transcription factors (GRMZM2G070211 and GRMZM2G25430), four of HB transcription factors (GRMZM2G023291, GRMZM2G097349, GRMZM2G099319, and GRMZM2G119999), and so on. In contrast with the especially expressed transcription factors in the pericarp, 79 transcription factors were isolated with the increased expression level in the aleurone layer of Ha6130 (Fig. 7B). These genes belonged to 29 transcription factor families, which included eight of MYB transcription factor, three of MYB-related transcription factors, three of NAC transcription factors, four of bHLH transcription factors, and so on.

### Verification of unigenes and gene expression profiling using RT-qPCR

**Figure 8:**
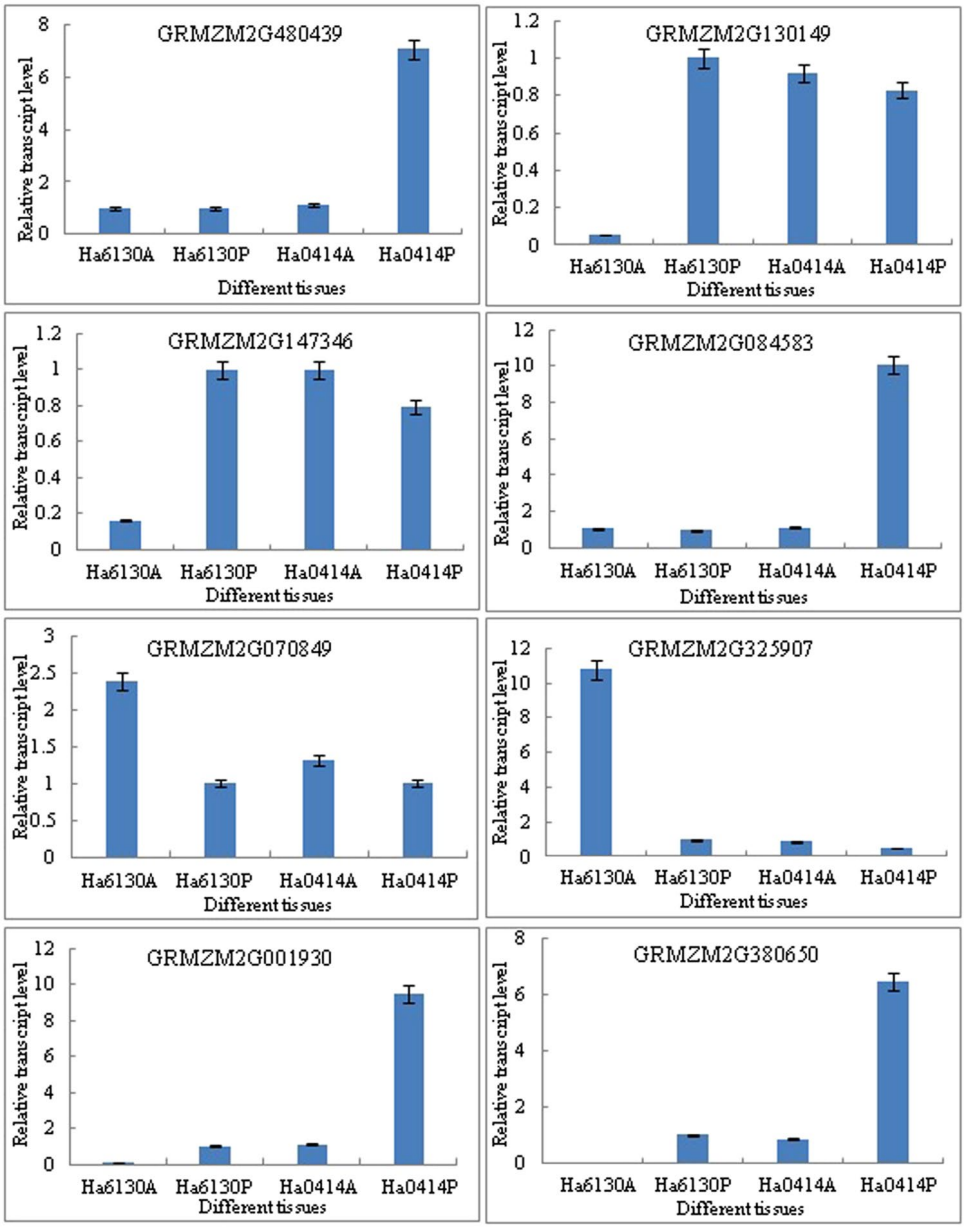
Verification of unigenes and gene expression profiling using RT-qPCR. Note: GRMZM2G480439: Glutathione S-transferase, GRMZM2G130149: R2R3-Myb domain protein, GRMZM2G147346: R2R3-Myb domain protein, GRMZM2G084583: R2R3-Myb protein, GRMZM2G070849: R2R3-Myb domain protein, GRMZM2G325907: R2R3-Myb domain protein. GRMZM2G001930: Transcription factor MYC7E, GRMZM2G380650: Chalcone synthase.

To evaluate the validity of sequence data, eight known transcripts were selected for examination by real-time RT-PCR. Information for these genes and their gene-specific primers are showed in Supplementary Table 3. The results showed that the expression patterns determined using both RT-qPCR and DGE were consistent for eight genes (Fig. 8), which suggests that the transcriptome analyses were very reliable.

## Discussion

Corn containing anthocyanins is one of the excellent sources of antioxidant compounds. In contrast with non-colored corn and yellow corn, purple corn contains more anthocyanins and shows favorable biological functions for body health such as antioxidant, antimicrobial, antiobesity, and so on^17–19^. It was verified to be a potential preventive agent for the treatment of diabetes-associated glomerulosclerosis accompanying proteinuria and kidney filtration dysfunction^2,13,18^. In corn, anthocyanins were usually deposited in the pericarp and aleurone layer of seed^1,6,20^. In the experiment, we identified two purple corn inbred lines with anthocyanins individually accumulated in the pericarp and aleurone layer, respectively.

In colored maize, the structure and concentration of anthocyanin components confer the color the seeds by expression of red, purple and blue^21^. For purple corn, the individual anthocyanins have been identified, which mainly contains cyanidin-3-glucoside, pelargonidin-3-glucoside, peonidin-3-glucoside, and their malonate derivatives. Nevertheless, in waxy corn, the most dominant components are the cyaniding-3-glucoside and its derivatives including cyanidin-3-glucoside, pelargonidin-3-glucoside, peonidin-3-glucoside, cyanidin-3-(6″-malonylglucoside), pelargonidin-3-(6″-maloylglucoside), and other components^22^. Furthermore, some individual anthocyanins components are different among the distinct genotypes during the mature stage^22^. In the experiments, the cyanidin-3-*O*-glucoside and pelargonidin-3-*O*-glucoside were verified to be the main components of anthocyanins in the pericarp of Ha0414 and aleurone layer Ha6130. Otherwise, the peonidin-3-glucoside was only characterized in the pericarp of Ha0414. Besides, between the pericarp and aleurone layer, the quantitative accumulations of anthocyanins are different. Generally, total monomeric anthocyanins in pericarp were ten times more than that in the aleurone layer. The maximum anthocyanins were present in the pericarp^23^. Similarly, total anthocyanins content in the pericarp of Ha0414 was more than that in the aleurone layer of Ha6130. On the contrary, the ear shading treatment demonstrated that anthocyanins content in the pericarp of Xixingchinuo NO. 1 is lower than that in the aleurone layer of Xixingheinuo NO. 1^20^. So, the accumulations of anthocyanins are different between the pericarp and aleurone layer. The level of anthocyanins content may depend on germplasm sources.

In maize, most pathway genes of anthocyanins encoding PAL, CHS, CHI, F3H, DFR, ANS, UFGT, and GST have been identified^15,16^. In the experiment, 16 genes identified in the pericarp of Ha0414 and 14 genes isolated in the aleurone layer of Ha6130 were mapped to the biosynthetic pathway of anthocyanins. Among these genes, two genes in the pericarp of Ha0414 (GRMZM2G162158 and GRMZM2G703582) and one gene in the aleurone layer (GRMZM2G703582) encoding 2-oxoglutarate (2OG) and Fe (II)-dependent oxygenase superfamily proteins were isolated with increased transcript abundance, which has the same domains with F3H and ANS^24^. Similarly, another gene encoding UDP-glycosyltransferase superfamily protein (GRMZM2G066067) showed especially increased expression level in both the pericarp of Ha0414 and the aleurone layer of Ha6130. Otherwise, one gene encoding flavonoid 3′, 5′-hydroxylase (GRMZM2G089528), and another gene annotated as flavonoid 3′-monooxygenase (GRMZM2G085845) only exhibited increased transcript abundance in the aleurone layer of Ha6130. The two genes have the similar function with F3′H. Besides, transcription factors participated in the regulation of anthocyanin biosynthesis. And two sets of duplicated genes: *booster1* (*b1*)/*red1* (*r1*), the class of bHLH transcription factors, and *colorless*1 (*c1*)/*purple plant1* (*pl1*), members of the R2R3-MYB family were both involved in the control of anthocyanin biosynthesis^11^. *B/Pl* confers the color of the pericarp. *R1/C1* is required for pigmentation of the aleurone layer^15,25^. *Purple aleurone1* (*Pr1*) regulates the accumulation of pelargonidin in aleurone layer under the control of *c1* and *r1* ^26,27^. In the experiment, three of bHLH transcription factors were isolated with increased transcript abundance in the pericarp of Ha0414, which was similar with b1 and r1. Besides, two of WRKY transcription factors, four of HB transcription factors and other transcription factors were also identified with increased transcript abundance in the pericarp of Ha0414. In addition, eight of MYB transcription factors, three of MYB-related transcription factors, three of NAC transcription factors, four of bHLH transcription factors, and other transcription factors were isolated with increased expression level in the aleurone layer of Ha6130. These genes’ specific expression may directly result in the tissue-specific biosynthesis of anthocyanins in pericarp and aleurone layer.

## Materials and Methods

### Plant material

Two purple corn lines Purple 1 and Purple 2 were isolated from preserved breeds in rural areas of Anhui province, People’s Republic of China. Anthocyanins were accumulated in aleurone layer for Purple 1 line and deposited in pericarp for Purple 2 line. By hybridization with the combination Purple 1 × Purple 2, the plants with anthocyanins accumulation in aleurone layer or pericarp were selected to generate inbred lines by continuous self-pollination. Then, two purple corn lines Ha0414 and Ha6130 were obtained for their similar color in the seeds from an F7 generation in the year 2015. They were cultivated with normal water and fertilizer management in the farm of Tobacco Research Institute. After pollination, the seeds were collected at 5, 10, 15, 20, 25, 30, 35 and 40 days post-pollination (DPP). On the one hand, the seeds of 25 DPP were prepared for the microscopic observation; on the other side, the pericarp and aleurone layer was peeled from the seeds of 25 DPP and stored at −70 °C for RNA extraction and genes expressions analysis.

### Microscopic observation of anthocyanins deposition

Seeds of 25 DPP stage were collected and immersed in FAA fixative (formaldehyde: 50% ethanol: 1% acetic acid = 5:90:5, v- v- v), then subjected to a light vacuum until the seeds sank^28^. After fixed overnight at 4 °C, seeds were rinsed with 100% ethanol for three times, and then embedded in paraffin blocks. By using a microtome, 14–16-μm-thick cross sections were obtained to observe the cross-section of the seed. Moreover, by using anatomical lens and tweezers, the fresh pericarp and aleurone layer were dissected and separated from the seed at 25 DPP stage for the record of the front side of aleurone layers and pericarp. The microstructure was observed under the microscope and documented by using the digital camera (Leica CTR6000, Germany).

### Anthocyanins content determination

To determine the anthocyanins content, 1 g of fresh seeds were collected at 10, 20, 30 at 40 DPP stages, then homogenized in 25 ml of an acid-ethanol solution (95% ethanol: 10% acetic acid: 0.1% hydrochloric acid = 95: 4: 1, v- v- v)^29^. After ultrasonic vibration at 25 °C for 30 min, the mixture was centrifuged at 6000 × g for 10 min. The supernatants were collected, and the absorbance readings were recorded at 535 nm using a photodiode array spectrophotometer (SP-752PC, Shanghai Spectral Instrument Company, China). The cyanidin-3-glucoside was selected as standard to evaluate the content of anthocyanins^20^.

### Anthocyanin components analysis by high-performance liquid chromatography

The dried mature seed was selected and ground into flour. Then, 5 g sample was dissolved in 25 ml of a mixture (100% ethanol: water: 0.1% hydrochloric acid = 2:1:1, v- v- v) and fully mixed with ultrasonic vibration at 25 °C for 30 min. The mixture was subsequently centrifuged at 8000 × g for 10 min. At last, the supernatants were collected after filtered with a 0.2 μm nylon membranes (Millipore, Bedford, MA). For further analysis, the Agilent model 1100 equipment was used and equipped with a Hypersil ODS C18 column (250 mm × 4.6 mm × 5 μm). The analysis was performed using 1% formic acid in water (Solvent A) and 1% formic acid in acetonitrile (Solvent B) as solvents with gradient elution (Supplementary Table 2). The samples were filtered through a 0.45 μm syringe filter (Millipore, Bedford, MA) before injection into the HPLC. Chromatographic profiles of anthocyanins were recorded at 520 nm. Cyanidin-3-glucoside, pelargonidin-3-glucoside, peonidin-3-glucoside, petunidin-3-glucoside, malvidin 3, 5-diglucoside and delphinidin-3-glucoside were selected for the references.

### Total RNA Extraction for RNA-Seq and qRT-PCR

300 mg of the aleurone layer and pericarp was collected for the preparation of the RNA by using RNAprep Pure Plant Kit (Tiangen, China). The quality and concentration of RNA were determined using NanoDrop 2000 (Thermo, USA). The RNAs were independently collected twice, creating two biological replicates.

### cDNA library construction and Sequencing

Library construction, quality detection, and Illumina sequencing were carried out by using Illumina TruSeq™ RNA Sample Preparation Kit (Illumina, San Diego, USA) at Beijing Novogene Biological Information Technology Co. Ltd. (Beijing, China) (HTTP://www.novogene.cn/). By using the TruSeq PE Cluster Kit v3-cBot-HS (Illumina), the index-coded samples were clustered following the manufacturer’s instructions. After cluster generation, the library was sequenced on an Illumina Hiseq. 2500 platform with 200 bp paired-end reads.

### Data filtering and assembly

By removing reads including adaptor sequences, duplicated sequences, ploy-N (reads with unknown nucleotides) and low-quality reads, high-quality clean reads were obtained, which were used for the all the downstream analyses. The resulting reads were aligned to a reference genome of the B73 reference sequence (AGPv2) and then generates the final transcriptome assembly using TopHat 2 according to Kim’s methods^30,31^.

### Analysis of differential expression genes

The procedure was performed according to standard digital genes expression (DGE) methods as described by Zhang *et al*.^32^. The read counts were adjusted by edger program package through one scaling normalized factor. Differentially expressed genes were screened by using the DEGSeq R package (1.12.0; TNLIST, Beijing, China). The P-values of 0.005 was set as the threshold for significant differential expression according to the Benjamini and Hochberg method^33^.

### Gene functional annotation and metabolic pathway analysis

The gene function was annotated using BLASTx (E-value <10^−5^) queries searched against four databases containing NCBI non-redundant protein database (Nr), Pfam (annotated Protein family) and SwissProt protein database. Then, by using the Blast2Go program, the genes were annotated according to molecular function, biological process, and cellular component ontology. Metabolic pathway assignments were performed using the Kyoto Encyclopedia of Genes and Genomes Pathway database (KEGG http://www.genome.jp/kegg). Moreover, to identify differentially expressed genes related to the tissue-specific accumulation of anthocyanins, the MapMan tool was used to isolate the target genes by mapping the genes to the biosynthetic pathway of anthocyanins according to the methods described by Kakumanu *et al*.^34^.

### Relative transcript level analysis

Both purple corn lines Ha0414 and Ha6130 were selected. Their pericarp and aleurone layer were respectively peeled from the seeds at 25 DPP and stored at −70 °C for RNA preparation and genes expressions analysis. Real-time RT-PCR was carried out using an SYBR Green assay (Takara Dalian, China) on an ABI PRISM® 7500 Sequence Detection System (Applied Biosystems, Foster City, USA). Each 20 µl assay contained 10 μl SYBR^®^ Premix Ex Taq™ II (2×), 2 μl cDNA and 100 nM of primer. The maize *GAPDH* gene was selected as the endogenous control. All primers were listed in Supplementary Table 3. The relative mRNA abundance was calculated according to the 2^−ΔCT^ method^35^.

## Supplementary information

Supplementary Tables and Figure

## Data Availability

The data supporting the findings of this study are available within the article and its Supplementary Information files. All other relevant source data are available from the corresponding author upon reasonable request.
