# Author Correction: Comparative transcriptome analysis reveals differentially expressed genes related to the tissue-specific accumulation of anthocyanins in pericarp and aleurone layer for maize

**DOI:** 10.1038/s41598-022-09965-5

**Published:** 2022-04-11

**Authors:** Tingchun Li, Wei Zhang, Huaying Yang, Qing Dong, Jie Ren, Honghong Fan, Xin Zhang, Yingbing Zhou

**Affiliations:** 1 Corn Research Center, Tobacco Research Institute, Anhui Academy of Agricultural Sciences, Hefei, 230031 People’s Republic of China; 2 School of Life Sciences, Anhui Agricultural University, Hefei, 230036 People’s Republic of China

Correction to: *Scientific Reports* 10.1038/s41598-018-37697-y, published online 21 February 2019 

This Article contains errors.

The Article states throughout that the gene GRMZM2G089528 is annotated as flavonoid 3’, 5’-hydroxylase or one of members of CYP706A protein. It should state that the gene GRMZM2G089528 is annotated as predicted flavonoid 3’, 5’-hydroxylase or one of members of CYP706A protein.

Two references were omitted and should be cited in the Introduction as Ref 36 and 37 as below:

“Corn (*Zea mays*) is one kind anthocyanins-rich source in cereals^2,11^.”

should read:

“Corn (*Zea mays*) is one kind anthocyanins-rich source in cereals^2,11, 36, 37^.”

These references are cited below as Reference 36 and 37, respectively.

Additionally, the Data Availability section in the original version of this Article was omitted.

It now appears as below:

**Data availability**

Raw RNA-sequencing data generated for this study is deposited in the NCBI Short Read Archive with the accession code PRJNA548440.

Finally, the lists of DEGs and transcription factors generated in this study are included in the Supplementary Information files included with this notice (Supplementary Tables 1 and 2, and Supplementary Table 3 and 4, respectively), as are FASTA files with DEG sequences.

## Supplementary Information

Supplementary Table 1.

Supplementary Table 2.

Supplementary Table 3.

Supplementary Table 4.

Supplementary Information.
