# Supplementary material for: Comparative transcriptome analysis reveals differentially expressed genes related to the tissue-specific accumulation of anthocyanins in pericarp and aleurone layer for maize
Source: Sci Rep. 2019 Feb 21;9:2485. doi: 10.1038/s41598-018-37697-y (PMC6384890; doi:10.1038/s41598-018-37697-y)
Supplement: Supplementary file 1 — Supplementary Tables and Figure [file 41598_2018_37697_MOESM1_ESM.docx]

**Comparative transcriptome analysis reveals differentially expressed genes related to the tissue-specific accumulation of anthocyanins in pericarp and aleurone layer for maize**

Tingchun Li^1, *^, Wei Zhang^1, 2^, Huaying Yang^1^, Qing Dong^1^, Jie Ren^1^, Honghong Fan^2, *^, Xin Zhang^2, *^, Yingbing Zhou^1, *^

**Supplementary Tables and Figure**

Supplementary Table 1 Genes involved in anthocyanin biosynthesis in maize

Supplementary Table 2 The mobile phase of HPLC

Supplementary Table 3 Primers for Real-time RT-qPCR

Supplementary Fig. 1 The correlation of genes expression between the aleurone layer and pericarp for Ha6130 and Ha0414

Supplementary Table 1 Genes involved in anthocyanin biosynthesis in maize

| Gene name | Encoding protein | Gene accession number |
| --- | --- | --- |
| Colorless2 (c2） | Chalcone synthase | 100274415 |
| chi1 | Chalcone isomerase | 100276821 |
| pr1 | Flavonoid 3'-hydroxylase | 100273383 |
| fht1(F3H) | Flavanone 3-hydroxylase | 542712 |
| a1 | Dihydroflavonol 4-reductase | 100286107 |
| anthocyaninless 2 (a2) | Anthocyanidin synthase | 100127010 |
| Bronze 1 (bz1) | UDP glucose flavonol 3*-O-*glucosyl transferase | 732800 |
| Bronze 2 (bz2) | Glutathione*-S-*transferase | 100502255 |
| ZmMrp4 | Multidrug resistance-like transporter | 100125659 |
| Sn | bHLH | X60706 |
| c1 | R2 R3-MYB | 100281156,100285289,541757,103643927,542612 |
| Pericarp color 1(p1) | R2 R3-MYB | 542272 |
| purple plant (pl) | R2 R3-MYB | 732799 |
| seed color component at R1 (r1) | bHLH | 100126972 |
| colored plant 1 (b1) | bHLH | 103646088 |
| pale aleurone color 1 (pac1) | WD40 | 100285351,103627556 |

Supplementary Table 2 The mobile phase of HPLC

| Time (min) | Flow rate (ml/min) | Solvent A (%) | Solvent B (%) |
| --- | --- | --- | --- |
| 0 | 0.8 | 92 | 8 |
| 2 | 0.8 | 88 | 12 |
| 5 | 0.8 | 82 | 18 |
| 10 | 0.8 | 80 | 20 |
| 12 | 0.8 | 75 | 25 |
| 15 | 0.8 | 70 | 30 |
| 18 | 0.8 | 55 | 45 |
| 20 | 0.8 | 20 | 80 |
| 22 | 0.8 | 92 | 8 |
| 30 | 0.8 | 92 | 8 |

Supplementary Table 3 Primers for Real-time RT-qPCR

| Genes | sequences Primer sequence (5’-3’) |
| --- | --- |
|  |  |
| GRMZM2G480439 | FP:TTCTCAGTTGGCTCCCGTTC RP:CTCAACAGTCCGGGAGATCG |
| GRMZM2G130149 | FP:TGGCTATCCCATGGACCAG RP:TTCCCAGACAGTTGGTGGTG |
| GRMZM2G147346 | FP:CTAGGATGCAGGATCGGAAGC RP:CACCCGACCGAGTTTCTCAG |
| GRMZM2G084583 | FP:AACCAAGCTGAACGAACGTC RP:CGTGCGTGTTCCAGTAGTTC |
| GRMZM2G070849 | FP:CGGACACAAGCAGTACGTCA RP:TACTGTACGCCGTCGTCTTG |
| GRMZM2G325907 | FP:CGCACCCCTTTTCCACTTTG RP:TTGGATGCTTACGGCCAAGA |
| GRMZM2G001930 | FP:GTCCTATCCGGTGGACGATG RP:ACCCCTCAATCCATGTGCTG |
| GRMZM2G380650 | FP:GGCGGTGCTTCAACTTCTTG RP:ACCATTACCCTGGCGGAATC |
| ZmGAPDH (M18976) | FP:CCCTTCATCACCACGGACTAC RP:AACCTTCTTGGCACCACCCT |
|  |  |


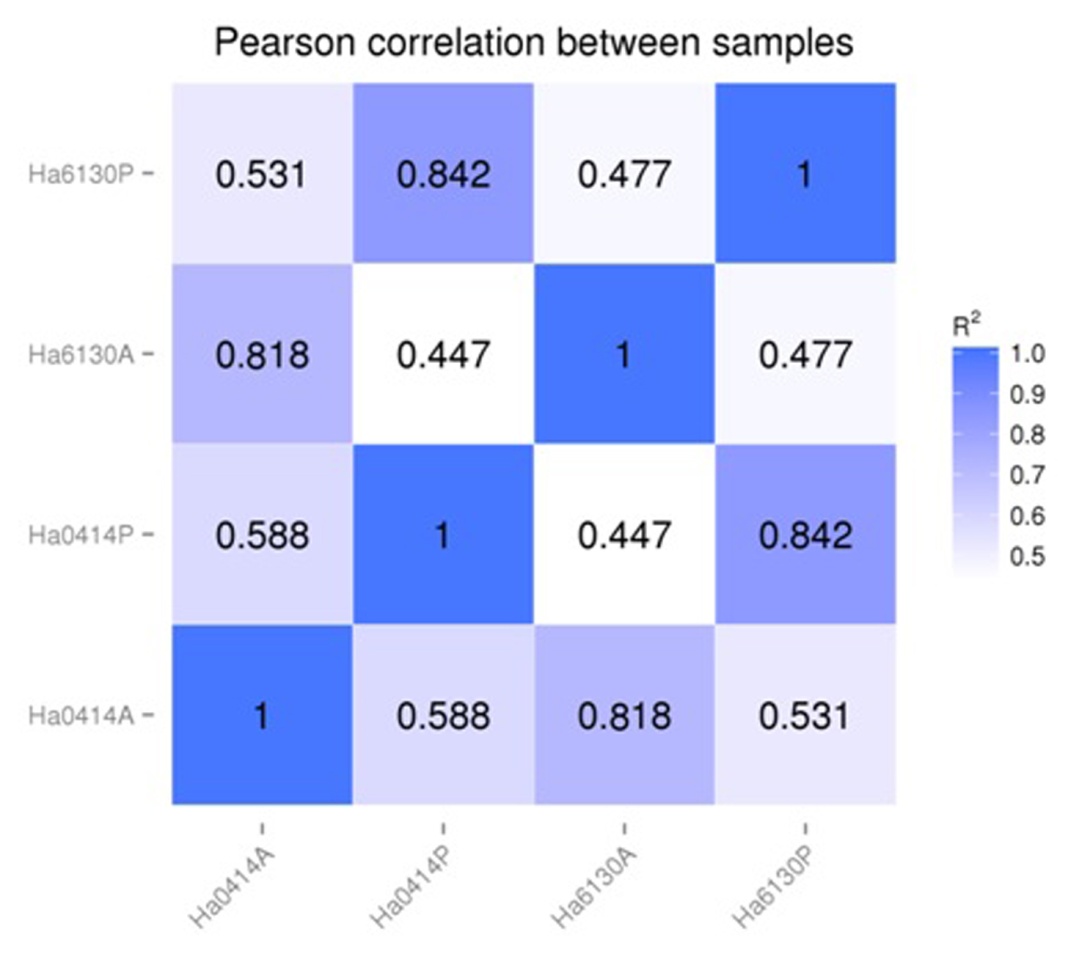


Supplementary Fig. 1 The correlation of genes expression between the aleurone layer and pericarp for Ha6130 and Ha0414
